# Aerosol Performance and Stability of Liposomes Containing Ciprofloxacin Nanocrystals

**DOI:** 10.1089/jamp.2015.1241

**Published:** 2015-12-01

**Authors:** David Cipolla, Huiying Wu, Igor Gonda, Hak-Kim Chan

**Affiliations:** ^1^ Department of Pharmacuetical Sciences, Aradigm Inc., Hayward, California.; ^2^ Faculty of Pharmacy, University of Sydney, Sydney, New South Wales, Australia.

**Keywords:** aerosol, ciprofloxacin, liposomes, nanocrystals, nebulization

## Abstract

***Background:*** Previously we showed that the release properties of a liposomal ciprofloxacin (CFI) formulation could be attenuated by incorporation of drug nanocrystals within the vesicles. Rather than forming these drug nanocrystals during drug loading, they were created post manufacture simply by freezing and thawing the formulation. The addition of surfactant to CFI, either polysorbate 20 or Brij 30, provided an additional means to modify the release profile or incorporate an immediate-release or ‘burst’ component as well. The goal of this study was to develop a CFI formulation that retained its nanocrystalline morphology and attenuated release profile after delivery as an inhaled aerosol.

***Methods:*** Preparations of 12.5 mg/mL CFI containing 90 mg/mL sucrose and 0.1% polysorbate 20 were formulated between pH 4.6 to 5.9, stored frozen, and thawed prior to use. These thawed formulations, before and after mesh nebulization, and after subsequent refrigerated storage for up to 6 weeks, were characterized in terms of liposome structure by cryogenic transmission electron microscopy (cryo-TEM) imaging, vesicle size by dynamic light scattering, pH, drug encapsulation by centrifugation-filtration, and *in vitro* release (IVR) performance.

***Results:*** Within the narrower pH range of 4.9 to 5.3, these 12.5 mg/mL liposomal ciprofloxacin formulations containing 90 mg/mL sucrose and 0.1% polysorbate 20 retained their physicochemical stability for an additional 3 months refrigerated storage post freeze-thaw, were robust to mesh nebulization maintaining their vesicular form containing nanocrystalline drug and an associated slower release profile, and formed respirable aerosols with a mass median aerodynamic diameter (MMAD) of ∼3.9 μm and a geometric standard deviation (GSD) of ∼1.5.

***Conclusions:*** This study demonstrates that an attenuated release liposomal ciprofloxacin formulation can be created through incorporation of drug nanocrystals in response to freeze-thaw, and the formulation retains its physicochemical properties after aerosolization by mesh nebulizer.

## Introduction

Historically, treatment of many lung diseases involved systemic delivery of the drug; however, this modality was generally replaced by inhalation that is more effective due to targeting drug to the site of disease and safer due to reduced systemic side-effects.^(1–4)^ Inhalation delivery provides effective therapy for a number of lung diseases where the drug operates at the site of disease [e.g., cystic fibrosis, asthma, and chronic obstructive pulmonary disease (COPD)].

For many classes of inhaled drug products, there are a variety of inhalation delivery technologies, including metered dose inhalers (MDIs), dry powder inhalers (DPIs), and soft-mist inhalers and nebulizers that provide preference options to the end user, the patient.^(5)^ However, most of the formulations in these inhaled products are limited in their duration of action due to rapid clearance from the lung.^(6)^

Sustained release inhaled formulations have the potential to improve treatment efficacy by reducing the frequency of dosing and providing therapeutic drug levels over a longer period of time.^(6)^ While no sustained release inhaled products have been approved, liposomal formulations are in the advanced stages of development^(6–8)^ with two products in late phase clinical trials: liposomal amikacin^(9–11)^ and liposomal ciprofloxacin (CFI).^(12,13)^ A mixture of CFI and free ciprofloxacin, termed Pulmaquin^®^, is currently in Phase 3 trials in non-cystic fibrosis (CF) and bronchiectasis (NCFBE) and was found efficacious in Phase 2 trials in CF^(12)^ and NCFBE,^(13)^ and in preclinical models of nontuberculous mycobacteria (NTM)^(14)^ and biodefense applications.^(15–17)^

The drug release profile can be designed into a liposome formulation by judicious selection of its vesicle size and composition.^(18,19)^ However, once a liposomal composition is manufactured, until recently, there was no general method to further modify the drug release profile. In recent studies, the encapsulation and drug release properties of a liposomal ciprofloxacin formulation were modified to provide either faster release, through the addition of surfactant under hyperosmotic conditions,^(20)^ or slower release, by transformation of the drug into nanocrystalline form in response to freeze-thaw [Cipolla, unpublished data].

This transformation introduces a rate-limiting dissolution step prior to diffusion of drug across the vesicle bilayer. The presence of the nanocrystals causes the unilamellar liposomes of ∼90 nm size to form an elongated shape with a mean increase in vesicle size by dynamic light scattering (DLS) of 40–60 nm. The addition of 0.1 to 0.2% polysorbate 20 to the formulation prior to freeze-thaw reduces both the size of the nanocrystal, and thus the extent of liposome elongation with only a ∼5 nm increase in mean vesicle size [Cipolla, unpublished data]. The CFI liposomes containing 0.1% polysorbate 20 were chosen for these aerosolization studies because their more compact liposome vesicles were more likely to be robust to the nebulization process.

While hundreds of liposome formulations have been developed specifically for inhalation applications, only a small fraction are robust to the nebulization process with full retention of encapsulated drug and no change in vesicle size distribution.^(8)^ The inclusion of surfactant in a liposome formulation may increase the likelihood of vesicle disruption during nebulization, and jet nebulization in particular is to be avoided due to excessive foaming of the surfactant.^(21)^

As an example, a deformable liposome formulation containing polysorbate 80 was unstable to jet, ultrasonic, and vibrating mesh nebulization with release of up to 98% of the encapsulated salbutamol sulfate drug.^(22)^ Furthermore, there have not been any reports of a liposomal formulation containing drug nanocrystals being aerosolized. Thus, it might be expected that a liposomal formulation containing both surfactant and nanocrystals would be unstable to the nebulization process.

The goal of this study was to identify liposomal ciprofloxacin nanocrystalline formulations that were stable to mesh nebulization and retained their crystalline structure conferring slower release in an *in vitro* release (IVR) assay. These formulations would presumably possess long-term stability in the frozen state, and would likely be used by the end patient immediately after thawing and formation of the nanocrystalline structures. To test this hypothesis, samples were stored in the frozen state for up to 3 months and characterized after thawing. Furthermore, there was interest in whether the selected CFI formulations would be stable for an extended period of time post freeze-thaw. Thus, these formulations varying in pH between 4.6 and 5.9 were stored at room temperature for up to 6 weeks post freeze-thaw to evaluate the stability of their physicochemical properties.

## Materials and Methods

### Materials

CFI, a liposomal ciprofloxacin formulation comprised of 50 mg/mL ciprofloxacin in a pH 6.0 histidine-buffered aqueous formulation, was manufactured by Northern Lipids Incorporated (Burnaby, BC, Canada). (The ciprofloxacin concentrations are expressed in terms of ciprofloxacin hydrochloride.) The following materials were utilized: sodium chloride (Amresco, Solon, OH), sucrose (Sigma-Aldrich, St. Louis, MO), HEPES, free acid (Avantor, Center Valley, PA), polysorbate 20 (VWR Int., West Chester, NJ), HPLC grade methanol (Fisher Scientific, Fair Lawn, NJ), triethylamine (TEA, JT Baker, USA), Donor Adult Bovine Serum (HyClone, Logan, Utah), and Nanosep centrifugal filtration devices, 10K and 30K molecular weight (Pall Corporation, Ann Arbor, MI). Deionized water was used for all studies.

### Preparation of liposomal ciprofloxacin

CFI is an aqueous dispersion of unilamellar liposomes of ∼80–90 nm containing hydrogenated soy phosphatidylcholine (HSPC) and cholesterol. The preparation of CFI has been reported previously.^(20,21)^ Briefly, multilamellar liposomes are extruded through membranes to produce unilamellar liposomes that are then actively loaded with ciprofloxacin.^(23,24)^ Any unencapsulated ciprofloxacin is removed by diafiltration, resulting in >99% encapsulated ciprofloxacin at a target concentration of 50 mg/mL ciprofloxacin.

### Preparation and selection of CFI formulations

CFI was diluted from 50 mg/mL to 12.5 mg/mL with water, sucrose, and sodium acetate buffer (25 mM sodium acetate, 145 mM NaCl, pH 4.0). The target pH was 5.9 (for the unadjusted formulation), and 5.3, 4.9, and 4.6 for the three pH adjusted formulations by varying the proportion of water or sodium acetate buffer that was added. These four formulations containing 12.5 mg/mL CFI, 90 mg/mL sucrose, 0.1% polysorbate 20, but differing in pH, were stored frozen (-50°C) and characterized after thawing. Ten mL of each formulation was prepared and ∼1 mL aliquots were pipetted into ten 1 mL glass HPLC vials and frozen at −50°C until use.

At two time points, including 1 day and 3 months, vials were removed and thawed for analysis of their appearance, pH, vesicle size distribution, drug encapsulation, and IVR profile. The aerosol properties were determined at the initial time point. Once thawed, the formulations were stored at room temperature for 6 weeks to determine their short-term stability post freeze-thaw with respect to any changes in vesicle size, and for the pH 4.9 formulation, the drug encapsulation state and IVR profile as well.

### Aerosol particle size characterization by laser diffraction and cascade impaction

The aerosol particle size distribution of the 12.5 mg/mL CFI formulations containing 90 mg/mL sucrose and 0.1% polysorbate 20, were determined using laser diffraction (HELOS/BF, Sympatec GmbH, Clausthal-Zellerfeld, Germany) as described previously.^(21)^ Briefly, the aerosol output from the PARI eFlow rapid nebulizer (Pari Pharma, Starnberg, Germany) using a 4 μm mesh was drawn at 12.5 L/min through a flow-through cell in front of the optical lens, and twenty time slices of 500 μsec each were analyzed. WINDOX 5 software was used assuming a shape factor of 1.00, density of 1.019 g/cm^3^, and Mie analysis mode to calculate a volume mean diameter (VMD) and geometric standard deviation (GSD).

The mass median aerodynamic diameter (MMAD) and GSD were measured in duplicate Andersen Cascade Impaction (ACI) (Thermo Andersen, Pittsburgh, USA) runs for the 12.5 mg/mL CFI formulation containing 90 mg/mL sucrose, and 0.1% polysorbate 20, pH 4.9, before (unfrozen control) and after freeze-thaw (containing nanocrystals). Two mL aliquots of each formulation were loaded into the PARI eFlow^®^ rapid nebulizer reservoir. The nebulizer was turned on and the aerosol output was drawn into the ACI at a flow rate of 30 LPM. After the end of nebulization, each plate was washed with pH 4 acetate buffer to ensure solubilization of ciprofloxacin, diluted with methanol, and then assayed for ciprofloxacin content.

To calculate the MMAD and GSD values, the drug mass deposited in the induction port (throat) and the plate associated with stage 0 was not included due to the unavailability of a precise upper size limit for droplets deposited in those sections. Note that the compendial stage cut-off values were multiplied by 0.971 to reflect the change in flow rate from the compendial value of 28.3 L/min to the flow rate of 30.0 L/min used in these experiments.

### Drug encapsulation in discrete aerosol size fractions

In the ACI experiments described above, the formulation recovered from stages 2, 3, and 4 of the ACI (the stages with the most deposition) was also evaluated for drug encapsulation as described in the drug encapsulation method section. This experiment was performed to determine whether the ACI is an acceptable collection system to assess the integrity of the aerosolized control or nanocrystalline CFI formulations.

### Characterization of the formulation integrity after aerosolization

While compendial aerosol QC tests (e.g., ACI, Liquid Impinger (LI), or Next Generation Impactor (NGI)) are generally suitable for determination of the mass of drug in discrete particle size increments, these tests are often inappropriate to characterize the integrity of drug products that are sensitive to degradation due to exposure to shear or drying on the collection surfaces.^(25)^ In addition, sample dilution that occurs in the LI or NGI collection vessel or after washing of the ACI plates makes it untenable to conduct cryo-TEM imaging or IVR analysis of the aerosolized liposome vesicles using these methods because the samples are too dilute.

**FIG. 1.**
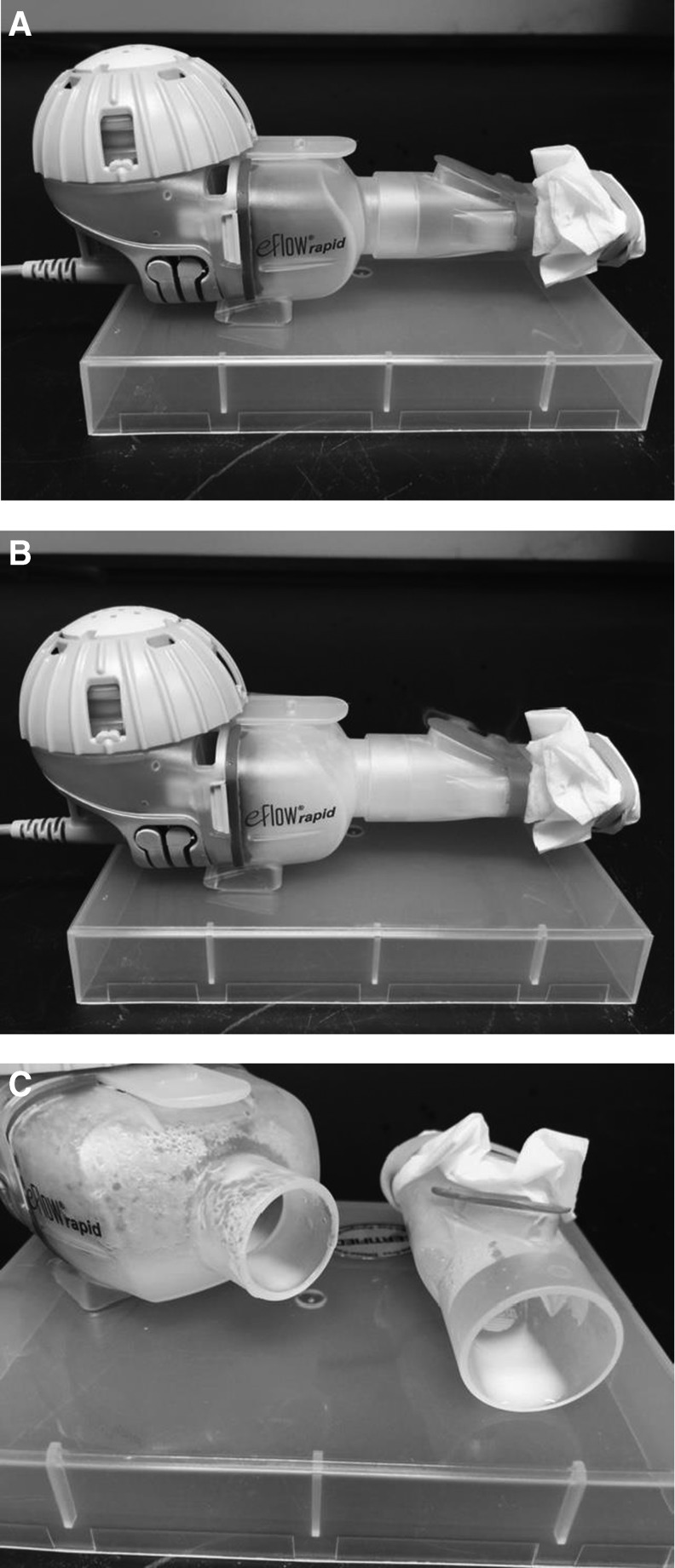
**(A)** PARI eFlow Rapid prior to nebulization; **(B)** PARI eFlow Rapid during nebulization showing aerosol entering the chamber and mouthpiece; **(C)** Deposited aerosol in the chamber and mouthpiece post nebulization.

Thus a simple aerosol collection procedure was utilized that allowed for an assessment of the effect of aerosolization on the state of the liposomes containing drug nanocrystals. Immediately after freeze-thaw, 1 or 2 mL aliquots of each experimental CFI formulation at pH 5.9, 5.3, 4.9, and 4.6, containing 90 mg/mL sucrose and 0.1% polysorbate 20 were loaded into the Pari eFlow^®^ rapid nebulizer reservoir. The nebulizer was turned on and the CFI formulation was drawn through the vibrating mesh to create the aerosol. A kimwipe was secured over the device mouthpiece to enhance deposition of the aerosol in the mouthpiece to ensure that an adequate sample volume was available for subsequent characterization of liposome integrity (Fig. 1). Undeposited aerosol droplets visibly escaped out of the mouthpiece expiratory valve and were vented into a fume hood. The mean vesicle size, state of encapsulation, cryo-TEM imaging, and IVR profiles (for a subset of the formulations) were determined before and after nebulization for the collected aerosol.

### pH

The pH of the experimental samples was measured with a Beckman Phi690 pH Meter (Fullerton, CA) using a Beckman Coulter combination pH electrode, Model 511275 (Fullerton, CA). Calibration was performed using Fisher (Fairlawn, NJ) pH 4.00 and 6.00 buffer standards.

### Vesicle size

The vesicle size distribution was measured using a Submicron Particle Sizer Autodilute Model 370 (Nicomp, USA). Each sample was diluted with isotonic saline to a concentration of ∼2 mg/mL liposomes and 0.5 mL was transferred to a disposable culture tube (Kimble Glass Inc., Vineland, NJ). The mean and standard deviation (SD) of the vesicle size distribution were recorded using the following instrument parameters: temperature: 23°C; viscosity: 0.933; refractive index: 1.333; intensity set point: 300 KHz; channel width: 10 μsec; scattering angle: 90; run time: 5 min; mode: vesicle; Gaussian distribution.

### Drug encapsulation

The percent drug encapsulation was determined by separating the free drug from the encapsulated drug using Nanosep centrifugation devices as described previously.^(26)^ Briefly, each sample was diluted ten-fold into acetate buffer (50 mM sodium acetate, 145 mM NaCl, pH 4.0) and 400 μL was transferred to the centrifuge device and centrifuged at 10,000 rpm (8100 *g*). The filtrate containing the free ciprofloxacin component was analyzed by HPLC to measure ciprofloxacin content. The percentage encapsulation of each sample was calculated by dividing the free drug component by the total drug content and multiplying by 100 to convert to a percentage. Results are reported as either single values or a mean of duplicate values.

### Cryogenic transmission electron aicroscopy (CryoTEM)

The 12.5 mg/mL CFI formulations at pH 4.9 and 5.3 stored refrigerated (control), after freeze-thaw in liquid nitrogen, and then after mesh nebulization, were evaluated by cryo-TEM imaging. A JEOL 2100 (Tokyo, Japan) instrument was operated at 200 kV to create cryo-TEM images of the liposome vesicles. Each sample was diluted with water to a liposome concentration of ∼10 mg/mL lipids, and 3 μL was transferred to a glow discharge Quantifoil carbon grid (Jena, Germany) in a chamber equilibrated at 22°C and 100% RH. Grids were blotted once with filter paper, at a blotting angle of 2 mm for 2 s, and vitrified by plunging into liquid ethane using a Vitrobot (F.E.I., Eindhoven, Netherlands). The vitrified samples were stored submerged in liquid nitrogen to retain sample integrity prior to cryoTEM analysis.

### In vitro *release (IVR)*

The IVR assay measures the release of encapsulated ciprofloxacin when incubated at 37°C in 50% bovine serum.^(26)^ Briefly, each experimental sample was diluted to 50 μg/mL ciprofloxacin in HEPES Buffered Saline (HBS: 20 mM HEPES, 145 mM NaCl, pH 7.4) and mixed one-to-one with chilled (2°–8°C) bovine serum and placed in a shaking water bath operated at 150 rpm and 37°C (Techne, TSBS40; Staffordshire, UK)). Duplicate samples were removed periodically (30, 60, 120, and 240 min), diluted 1:1 with chilled HBS buffer, and placed in an ice-water bath to terminate any further release of encapsulated drug from the liposomes. The released ciprofloxacin was separated from the liposome-encapsulated ciprofloxacin using the Nanosep centrifugal devices and quantified by HPLC as described previously.^(22)^ The percent release at each time point was determined by dividing the free drug by the total drug and multiplying by 100 to convert to a percentage. Results are reported as the mean with error bars representing the standard deviation of duplicate values.

While there is no statistical guidance to compare *in vitro* release profiles for liposome products, the Agency does recommend the use of similarity factor (f_2_) analysis to compare dissolution profiles for modified release solid oral dosage forms.^(27)^ To evaluate whether two IVR profiles are statistically different or similar, a comparison of the IVR profiles was made using this similarity factor (f_2_) analysis.^(27)^ The similarity factor was calculated by comparing the mean values of a test IVR profile to that of a reference IVR profile at each of the time points.^(27)^ An f_2_ value of 100 represents an identical test and reference profile. Similarity values (f_2_) greater than 50 are generally considered to indicate similarity, while an f_2_ value of less than 50 indicates that the profiles are different.^(23)^

### High performance liquid chromatography (HPLC)

An HPLC method was used to quantify the amount of ciprofloxacin in each sample as described previously.^(26)^ The separation and quantitation was conducted using a Nucleosil C-18 column (5 μm, 4.6 × 150 mm, Canadian Life Science, CA) with a Nucleosil C-18 guard column (4 × 3.0 mm, Phenomenex, USA) both at 35°C. The mobile phase was an isocratic mixture of 83:17 (v/v) of 0.5% TEA in water, pH 3.0, and 100% methanol. The elution was conducted at a flow rate of 0.9 mL/min. The content of ciprofloxacin was quantified using a wavelength of 277 nm. Ciprofloxacin concentrations were expressed in terms of ciprofloxacin hydrochloride.

## Results

### Stability in the frozen state for up to 3 months

#### Appearance, vesicle size, and drug encapsulation

Fifty mg/mL CFI was diluted with sucrose, polysorbate 20, acetate buffer (pH 4.0), and water to achieve 12.5 mg/mL ciprofloxacin, 90 mg/mL sucrose, 0.1% polysorbate 20, at pH values of 5.9, 5.3, 4.9, and 4.6. Samples of each formulation were thawed after either 1 day or 3 months frozen storage. After thawing, the visual appearance of every sample had good clarity. The mean liposome vesicle size for the unfrozen control was 89 to 90 nm by DLS (Table 1). For the four formulations ranging in pH between 4.6 to 5.9, the mean vesicle size increased to between 97 to 104 nm at the two time points following frozen storage at −50°C (Table 1), consistent with the formation of nanocrystals. There was no material difference in vesicle size as a function of pH or with respect to time stored frozen for any of the four formulations. The width of the vesicle size distribution for the freeze-thaw formulations increased compared to that for the unfrozen control, suggesting that the population of liposomes became more heterogeneous in size.

**Table 1.** Vesicle Size Distribution and Drug Encapsulation of 50 mg/mL CFI^a^

	*Mean vesicle size (nm) [SD]*^b^		*Encapsulation (%)*^b^	
	*Time (months)*		*Time (months)*	
*Formulation*	*0.03*	*3*	*0.03*	*3*
*Refrigerated*				
Control CFI	89.2 [27.0]	90.2 [23.6]	99.5	99.2
*Frozen*				
pH 5.9	98.5 [31.8]	99.6 [34.7]	82.2	80.9
pH 5.3	103.8 [37.1]	100.3 [31.4]	79.3	83.2
pH 4.9	103.4 [40.2]	96.7 [31.6]	77.2	83.6
pH 4.6	103.9 [38.1]	99.6 [30.7]	78.9	82.1

^a^ Control and pH adjusted 12.5 mg/mL CFI formulations containing 90 mg/mL sucrose, 0.1% polysorbate 20 after storage for 1 day (0.03 months) or 3 months.

^b^ Vesicle size data are reported as the mean (in nm) and standard deviation (SD). The encapsulation data are reported as the mean of duplicate analysis. The individual encapsulation values did not differ by more than 0.2% from the mean value.

The CFI control sample that was not frozen retained ∼99% drug encapsulation over the 3-month period during refrigerated storage (Table 1). In contrast, after freeze-thaw, the drug encapsulation decreased to around 80% for all of the 12.5 mg/mL CFI formulations, independent of the external pH (Table 1). The encapsulation values for the samples stored frozen for 3 months versus 1 day were also ∼80%, suggesting that ∼20% of the drug leaked out in response to the freezing or thawing process and that the duration of time the sample was in the frozen state had no impact (Table 1).

#### Cryo-TEM imaging

**FIG. 2.**
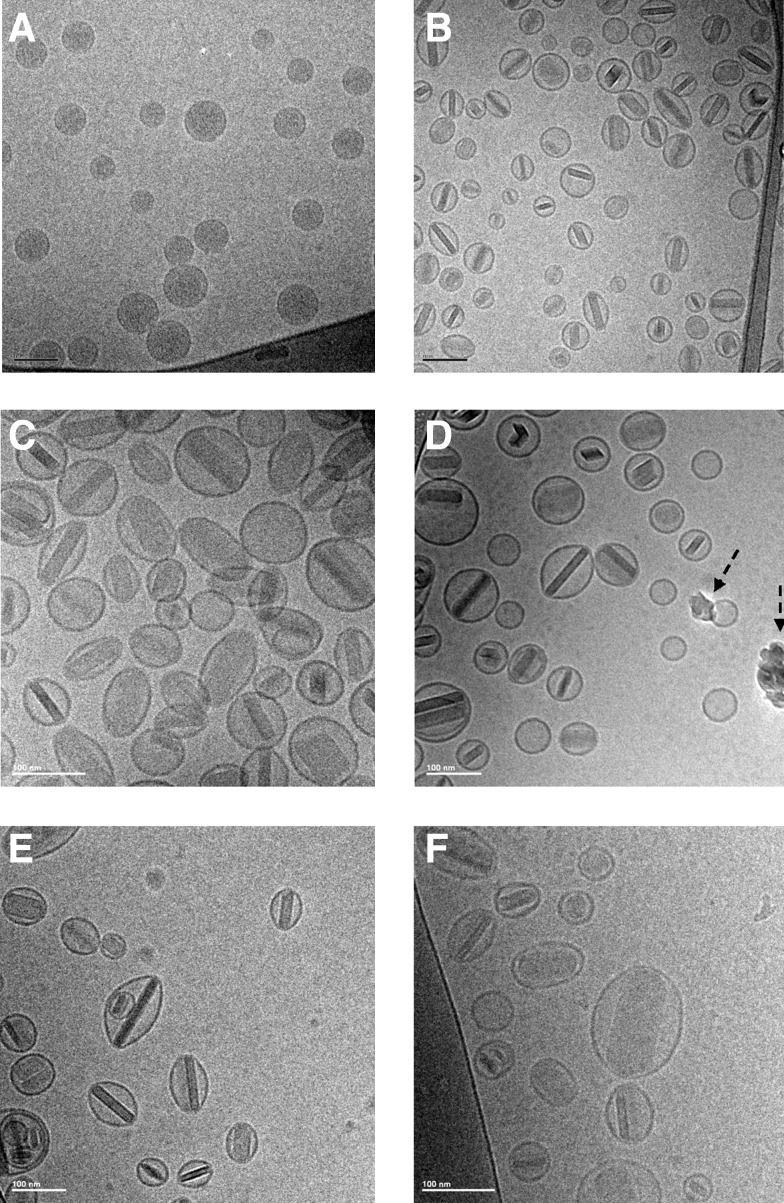
CryoTEM micrographs of 12.5 mg/mL CFI, 90 mg/mL sucrose, 0.1% polysorbate 20, before and after liquid nitrogen freeze-thaw, and after nebulization. The scale bar in the *bottom left-hand corner* is 100 nm for all images. All samples were applied at a concentration of ∼10 mg/mL liposomes. **(A)** Unfrozen control at pH 5.9; **(B)** pH 5.9 after thawing; **(C)** pH 5.3 after thawing; **(D)** pH 5.3 after thawing and nebulization; **(E)** pH 4.9 after thawing; **(F)** pH 4.9 after thawing and nebulization. The *dashed arrows* in **D** show ice artifacts introduced during sample preparation.

Prior to freeze-thaw, the liposomes in CFI are unilamellar with relatively uniform density consistent with encapsulation of ciprofloxacin (Fig. 2A). After freeze-thaw in liquid nitrogen, the cryo-TEM profiles of the three CFI formulations with pH values between 4.9 to 5.9 are comparable, each showing the presence of internalized drug nanocrystals (Fig. 2B, C, E). The range in vesicle size broadens due to heterogeneity in the size of the encapsulated nanocrystals. Liposomes with shorter nanocrystals retain a more circular profile in the cryo-TEM image, while liposomes containing longer nanocrystals have a more oblong shape.

#### IVR profiles

**FIG. 3.**
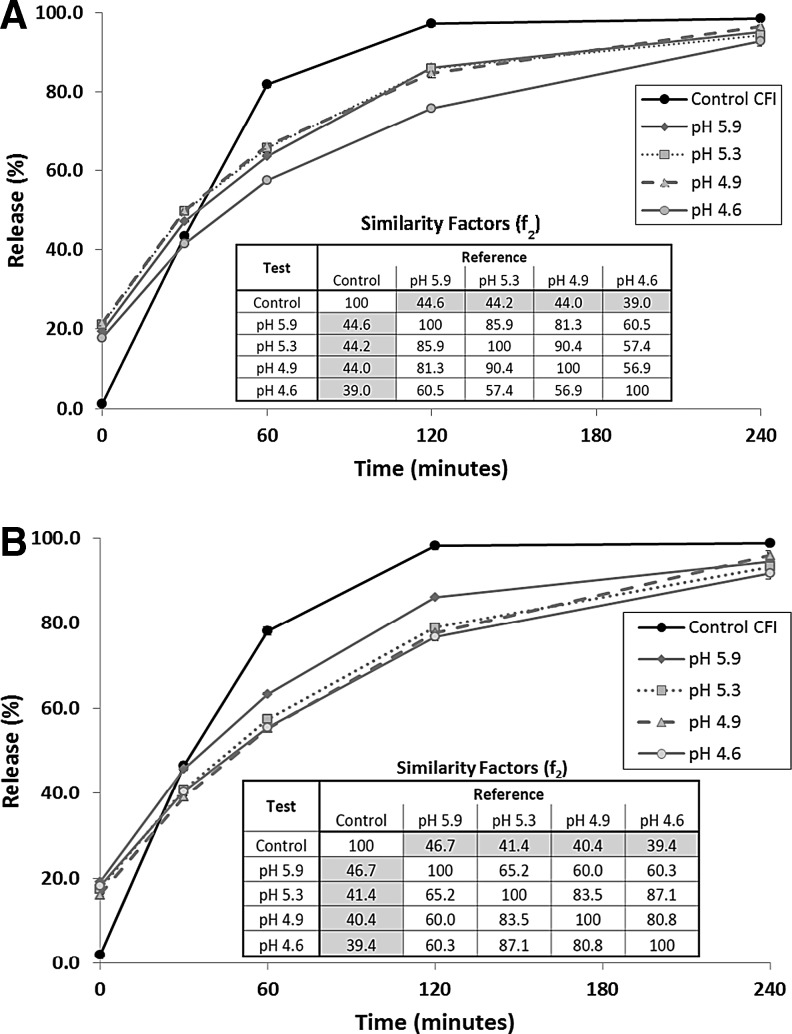
Evaluation of the effect of time in the frozen state on the IVR profile of CFI in 90 mg/mL sucrose and 0.1% polysorbate 20 at various pHs. **(A)** 1 day frozen; **(B)** 3 months frozen. Each CFI formulation was diluted to 50 μg/mL CFI in HEPES buffered saline (HBS) prior to a 1:1 dilution in bovine serum to measure the release of ciprofloxacin after incubation at 37°C for up to 4 h. IVR profiles for unfrozen control CFI (*black circles*), CFI at pH 5.9 (*gray diamonds*), CFI at pH 5.3 (*gray squares*), CFI at pH 4.9 (*gray triangles*), and CFI at pH 4.6 (*gray circles*). Error bars represent the standard deviation of duplicate values. The error bars that are not visible are smaller than the width of the data symbols.

After freeze-thaw, the IVR profiles of the four CFI formulations with pH values between 4.6 to 5.9 show similar characteristics to each other with an initial burst of approximately 20% compared to no burst for the unfrozen control (Fig. 3A). Not surprisingly, the IVR data at the T = 0 time point in the IVR assay is consistent with the encapsulation data (Table 1). The freeze-thaw formulations all have slower-releasing profiles compared to that for the unfrozen control (Fig. 3A). The inset in Figure 3A showing the f_2_ similarity factors indicates that all of the IVR profiles for the four freeze-thaw profiles are similar to each other, with similarity factors exceeding 50, and all are dissimilar to the unfrozen control, with similarity factors less than 50. The slower release profile for the formulations that were exposed to freeze-thaw is consistent with the presence of nanocrystals within the vesicles (Fig. 2B, C, E). The CFI formulation at the lowest pH value of 4.6 has a slightly slower release profile compared to the other three formulations but is still similar to those for the other pHs based on the f_2_ similarity test.

After 3 months frozen storage, the IVR profiles of the thawed formulations were comparable to those after only 1-day frozen storage (Fig 3B). They once again show an initial burst of ∼20% drug followed by a slower release compared to the unfrozen control. The pH 5.9 and 4.6 formulations stored frozen for 3 months essentially overlaid their profiles after 1-day frozen storage. In contrast, the pH 4.9 and 5.3 formulations had release profiles closer to that of the pH 5.9 formulation after 1-day frozen storage but after 3 months frozen storage were closer to that of the pH 4.6 formulation. The inset in Figure 3B showing the f_2_ similarity factors once again indicates that all of the IVR profiles for the four freeze-thaw formulations are similar to each other, with similarity factors exceeding 50, and all are dissimilar to that of the unfrozen control, with similarity factors less than 50.

### Aerosol characterization

#### Particle size distribution by laser diffraction and cascade impaction

Jet nebulizers were initially evaluated to aerosolize these CFI formulations containing nanocrystals but the presence of 0.1% polysorbate 20 caused foaming, which reduced the aerosol output rate and lengthened the nebulization time. Thus a vibrating mesh nebulizer, the PARI eFlow rapid with 4 μm mesh, was utilized. The aerosol particle size distribution of the four CFI nanocrystalline formulations was in a respirable size after mesh nebulization (Table 2). The volume mean diameter (VMD) ranged between 3.63 to 3.95 μm across the four formulations, with aerosol droplet size increasing slightly with decreasing pH. The aerosol geometric standard deviation (GSD) was ∼1.7 for all four formulations.

**Table 2.** Effect of Nebulization on Four pH-Adjusted 12.5 mg/mL CFI Formulations^*^

	*Mean vesicle size (nm) [SD]*		*Encapsulation (%)*		*Aerosol particle size distribution*	
*Formulation*	*Prior to nebulization*	*Collected aerosol*	*Prior to nebulization*	*Collected aerosol*	*VMD (μm)*	*GSD*
pH 5.9	98.4 [35.5]	109.4 [52.9]	82.6	78.9	3.63	1.65
pH 5.3	101.5 [29.7]	110.2 [46.1]	82.1	75.7	3.80	1.72
pH 4.9	102.9 [35.5]	108.2 [47.5]	84.9	76.8	3.88	1.71
pH 4.6	103.8 [39.2]	105.9 [45.5]	83.3	59.6	3.95	1.66

^*^ Formulations contain 90 mg/mL sucrose and 0.1% polysorbate. Vesicle size data are reported as mean (in nm) and [SD]. The state of ciprofloxacin encapsulation is reported in terms of percentage and represents single measurements. The aerosol particle size distribution is reported in terms of the Volume Mean Diameter (VMD) and Geometric Standard Deviation (GSD). SD, standard deviation.

**FIG. 4.**
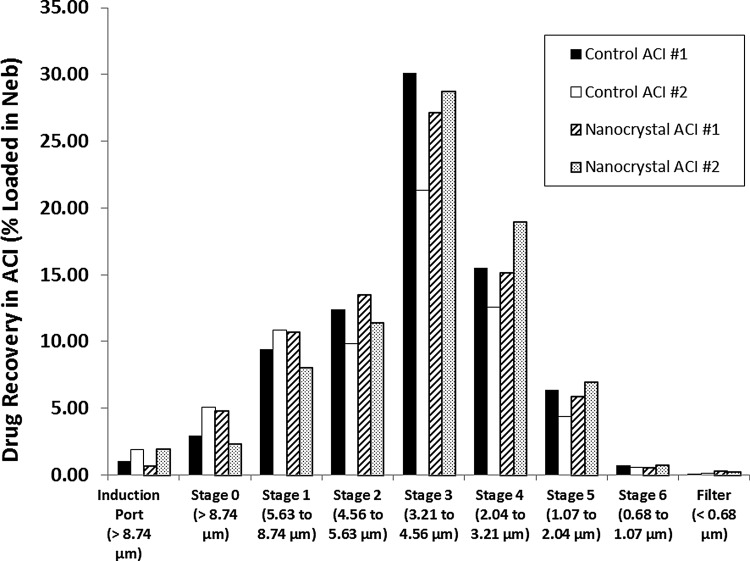
Cascade impaction profiles for nebulized 12.5 mg/mL CFI in 90 mg/mL sucrose and 0.1% polysorbate 20 at pH 4.9: unfrozen control (*black and white columns*) and after freeze-thaw in liquid nitrogen (*dashed and speckled columns*). ACI, Andersen Cascade Impactor.

When the aerosol of the unfrozen control pH 4.9 CFI formulation was characterized by ACI, the MMAD was ∼4.0 μm with a GSD of 1.45 (Table 3 and Fig. 4). The aerosol particle size distribution for the pH 4.9 nanocrystalline CFI formulation was comparable to that of the unfrozen control formulation with an MMAD ranging between 3.8 to 4.0 μm and a GSD of 1.45 (Table 3 and Fig. 4). The collected mass of drug in the ACI represented 67%–78% of the loaded nebulizer drug dose for the control CFI formulation and 79% of the loaded nebulizer drug dose for the nanocrystalline CFI formulation (Fig. 4).

**Table 3.** Drug Encapsulation State of 12.5 mg/mL CFI Formulation^*^

	Encapsulation (%)^**^			
	*CFI before freeze-thaw*		*CFI after freeze-thaw*	
*CFI prior to nebulization*	98		85	
*Sample*	*ACI #1*	*ACI #2*	*ACI #1*	*ACI #2*
ACI Stage 2	83	85	54	34
ACI Stage 3	85	90	64	27
ACI Stage 4	57	84	52	9

	*ACI #1*	*ACI #2*	*ACI #1*	*ACI #2*
Recovery in ACI (%)	78.3	66.7	78.7	79.1
MMAD (μm)	3.96	4.08	4.02	3.75
GSD	1.41	1.50	1.45	1.45

^*^ Formulation contains 90 mg/mL sucrose, 0.1% polysorbate 20, pH 4.9. Samples include: CFI samples before and after freeze-thaw, and then following nebulization and recovery from Stages 2, 3, and 4 of the ACI.

^**^ The encapsulation data are single measurements.

#### Formulation integrity after aerosolization: Vesicle size, encapsulation state, cryo-TEM imaging, and IVR profile

Using the PARI eFlow rapid, 1 mL of each formulation was nebulized and collected for analysis of integrity (Fig. 1). For the four experimental formulations, the mean vesicle size increased from between 98 to 104 nm prior to nebulization to 106 to 110 nm in the collected aerosol samples (Table 2). The ciprofloxacin encapsulation state of the four formulations varying in pH ranged between 82% and 85% prior to nebulization (Table 2). After nebulization, for the three formulations at the higher pH values, there was a relatively small drop in drug encapsulation to 76%–79% (Table 2). For the pH 4.6 formulation, there was a larger decrease in encapsulation state to ∼60% (Table 2). The cryo-TEM images for the pH 5.3 and 4.9 CFI formulations after mesh nebulization (Fig. 2D and 2F) are comparable to those prior to aerosolization (Fig. 2C and 2E) with retention of nanocrystalline structures within the liposome vesicles.

**Fig. 5.**
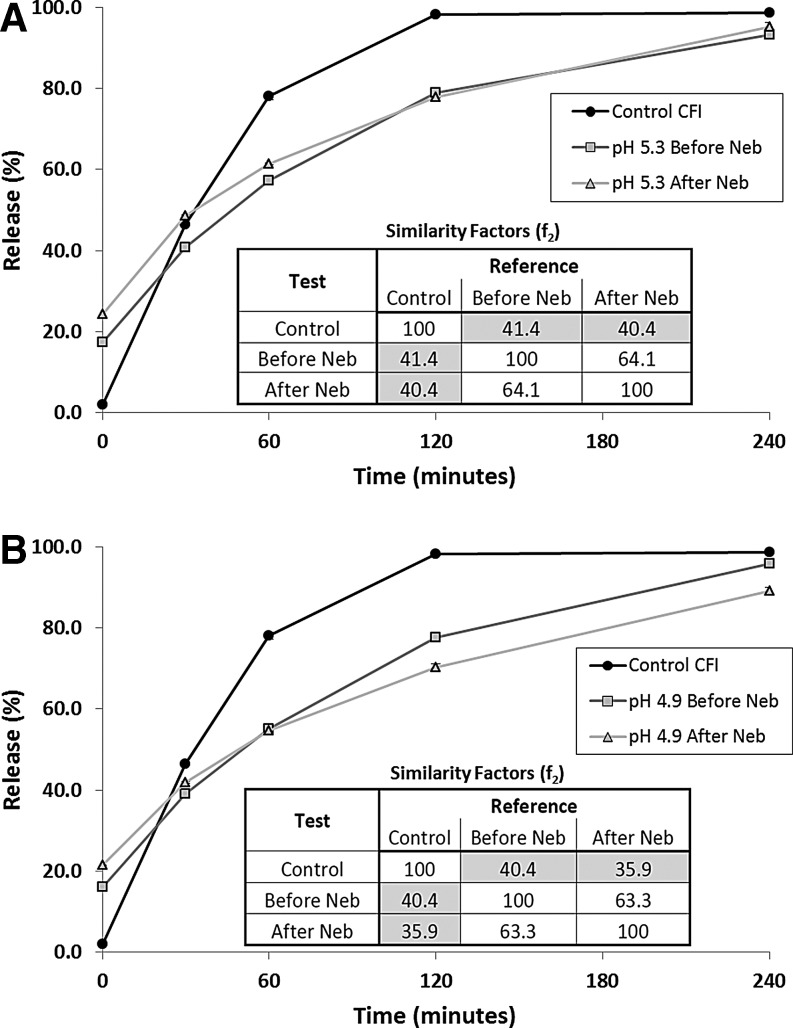
The effect of nebulization on the IVR profile. The control CFI formulation (at 50 mg/mL) and each of the 12.5 mg/mL CFI formulations were diluted to 50 μg/mL CFI in HEPES buffered saline (HBS) prior to a 1:1 dilution in bovine serum to measure the release of ciprofloxacin after incubation at 37°C for up to 4 h. **(A)** IVR profiles for Control CFI (*black circles*), CFI in 90 mg/mL sucrose, 0.1% polysorbate 20, pH 5.3, before nebulization (*gray squares*) or after nebulization (*gray triangles*). **(B)** IVR profiles for Control CFI (*black circles*), CFI in 90 mg/mL sucrose, 0.1% polysorbate 20, pH 4.9, before nebulization (*gray squares*) or after nebulization (*gray triangles*). The error bars that are not visible are smaller than the width of the data symbols.

The IVR profiles were characterized only for the two intermediate pH nanocrystalline formulations (4.9 and 5.3). After nebulization, the pH 5.3 nanocrystalline formulation possessed a slightly higher release value at the initial time point in the IVR assay (Fig. 5A), consistent with the small loss in drug encapsulation reported in Table 2. The pH 5.3 formulation retained its slow release profile over the course of the IVR assay, comparable to that for the formulation prior to nebulization (Fig. 5A). The inset in Figure 5A showing the f_2_ similarity factors indicates that the IVR profiles for the pH 5.3 formulation after freeze-thaw and then after nebulization are similar to each other, with similarity values exceeding 50, but are dissimilar to the unfrozen pH 5.3 control, with similarity factors less than 50.

The pH 4.9 formulation had a similar profile to the pH 5.3 formulation with just over a 20% initial burst in the IVR assay, followed by a slow release profile compared to the unfrozen control (Fig. 5B). The inset in Figure 5B showing the f_2_ similarity factors indicates that the IVR profiles for the pH 4.9 formulation after freeze-thaw and then after nebulization are similar to each other, with similarity values exceeding 50, but are dissimilar to the unfrozen pH 4.9 control, with similarity factors less than 50.

#### Drug encapsulation in discrete aerosol size fractions in the ACI

The previous aerosol characterization studies confirmed that while there were small changes in the vesicle size and encapsulation state of the pH 4.9 nanocrystalline formulation after mesh nebulization, these changes did not meaningfully affect the release profile (Fig. 5) or cryoTEM images (Fig. 2F). Characterization of the drug encapsulation state of the collected aerosol in the ACI was preformed to determine whether the ACI can also be used to assess the integrity of these liposomal formulations.

The drug encapsulation state was measured for both the unfrozen control CFI sample and the nanocrystalline CFI sample prior to nebulization, and then in the collected aerosol depositing on stages 2, 3, and 4 of the ACI as those contained the majority of the respirable aerosol (Table 3). For the unfrozen control formulation, collection in the ACI resulted in a large decrease in drug encapsulation from 98% prior to nebulization to 57%–90% for the collected aerosol on stages 2, 3, and 4 (Table 3). For the nanocrystalline liposome formulation, the ACI methodology introduced even greater vesicle disruption with drug encapsulation values dropping from 85% to between 9%–64% in the ACI (Table 3).

The nanocrystalline formulation was more affected by collection in the ACI, suggesting that the vesicles containing drug nanocrystals are more sensitive to the ACI collection methodology than the control vesicles. Stage 4, with the smallest deposition volume, but the largest number of discrete impaction points, and thus the greatest contact surface area to volume ratio, had the greatest loss in drug encapsulation for both formulations, suggesting that exposure to the air–liquid or solid-liquid interface and subsequent drying may be a key factor in vesicle disruption in the ACI. There was significant variability in the duplicate runs but further experiments to understand the sources of this variability were not conducted since it was determined that the ACI methodology introduced substantial artifacts during aerosol collection of these formulations.

### Short-term room temperature stability post freeze-thaw

The short-term stability of these nanocrystalline formulations in their thawed state was determined by storing them at room temperature (RT) conditions (23°–25°C) and periodically evaluating their appearance and vesicle size distribution. The formulation at pH 5.9 (of the four formulations, ciprofloxacin has the lowest solubility at this pH) was birefringent under optical microscope after 3 days, revealing the presence of crystals of ciprofloxacin external to the liposomes. All of the other nanocrystalline formulations retained good clarity over the 6 weeks of the study. For all four CFI formulations there was an initial increase in vesicle size of ∼10–15 nm over the first 10 days, but the mean vesicle size subsequently plateaued and remained stable out to 6 weeks at a vesicle size value which was only 5–10 nm larger than its initial value (Table 4).

**Table 4.** Vesicle Size at Room Temperature Storage after Freeze-Thaw of Four pH-Adjusted 12.5 mg/mL CFI Formulations^*^

	Mean vesicle size (nm)^**^					
	*Time (weeks)*					
*Formulation*	*0*	*0.4*	*1.4*	*2.3*	*3.4*	*5.3*
Unfrozen CFI	ND	89.2	ND	89.7	90.7	90.6
pH 5.9	98.5	104.0	109.8	106.6	109.0	104.8
pH 5.3	103.8	108.2	114.8	110.0	108.8	108.7
pH 4.9	103.4	110.0	117.5	111.1	112.8	111.6
pH 4.6	103.9	107.6	117.7	113.1	113.1	113.5

^*^ Formulations contain 90 mg/mL sucrose and 0.1% polysorbate 20.

^**^ The vesicle size data are reported as the mean (in nm). ND, not determined.

Only the pH 4.9 formulation was evaluated in experiments to determine whether the encapsulation state and release profile are affected by short-term storage at RT conditions. The encapsulation state of the pH 4.9 formulation stored for six weeks at RT conditions decreased to 79.7% (SD = 0.2%) relative to its initial value of 84.9% (Table 2). In this experiment the drug encapsulation state of a thawed vial of the original pH 4.9 formulation was measured on that day. It had an encapsulation value of 85.2% (SD = 0.3%) which is comparable to the original value of 84.9%.

**FIG. 6.**
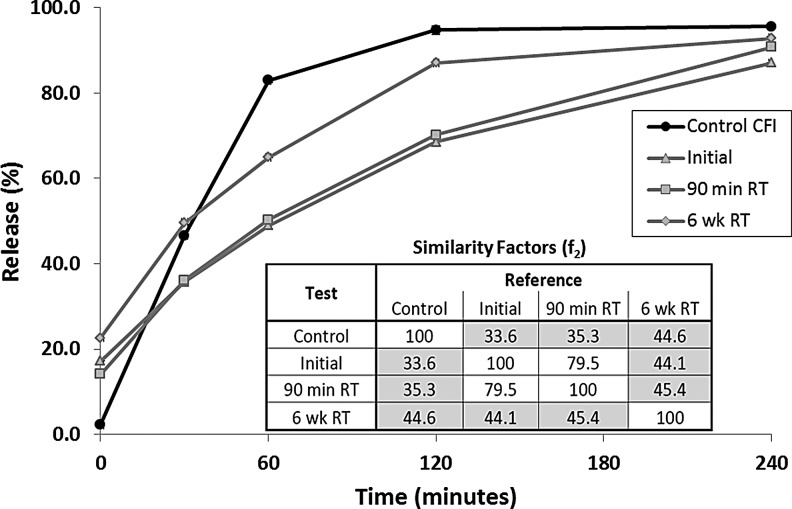
The effect of liposomal composition on the IVR profile. The control CFI formulation (at 50 mg/mL) and the 12.5 mg/mL CFI formulations were diluted to 50 μg/mL CFI in HEPES buffered saline (HBS) prior to a 1:1 dilution in bovine serum to measure the release of ciprofloxacin after incubation at 37°C for up to 4 h. The IVR profiles for Control CFI (*black circles*), CFI in 90 mg/mL sucrose, 0.1% polysorbate 20, pH 4.9, initially after freeze-thaw (*gray triangles*), after a 90 minute delay (*gray squares*), and after 6 weeks storage at RT (*gray diamonds*). The error bars that are not visible are smaller than the width of the data symbols. RT, room temperature.

The IVR profile of the pH 4.9 formulation was characterized immediately after freeze-thaw and then 90 minutes later to see if a 90-minute delay in use would lead to any impact on the release profile. There was no difference between the two profiles (Fig. 6) and the f_2_ similarity factors indicate that the IVR profiles were similar to each other (inset in Fig. 6). After 6 weeks storage at RT, the IVR profile was once again evaluated. There was an increase in the initial release at T = 0 consistent with loss of some encapsulated drug (Fig. 6). The overall release profile was intermediate between that of the nanocrystalline formulation immediately after freeze-thaw and that for the unfrozen control (Fig. 6). Thus, the nanocrystalline formulation retained some of its slow-release character even after 6 weeks RT storage. However, the IVR f_2_ similarity factors suggest that, while the formulation stored for 6 weeks at RT is still dissimilar to the unfrozen control, it is no longer similar to the formulation immediately after freeze-thaw.

## Discussion

We have investigated the potential to nebulize liposomal formulations containing ciprofloxacin encapsulated in a nanocrystalline state that confers a slower rate of drug release in an IVR assay. While there have been many liposomal formulations^(7–11,28,29)^ and nanocrystalline suspensions^(30,31)^ that have been nebulized, there have been no reports of successful nebulization of a liposomal formulation containing encapsulated nanocrystalline drug.

During nebulization, the formulation is exposed to shear and air–liquid interface, with both processes having the potential to disrupt liposomes, resulting in loss of drug and changes in the vesicle size distribution.^(8)^ Thus, it was not clear if liposomes containing nanocrystalline drug could withstand the added rigors of nebulization. The conversion of the encapsulated drug to nanocrystalline form, and the resulting stress on the liposomes to accommodate the long drug nanocrystals by assuming a more oblong shape, had the potential to reduce their ability to retain their structural integrity during the nebulization process.

The liposomal ciprofloxacin formulations without drug nanocrystals that formed the basis for these studies were previously shown to be robust to the nebulization process with no meaningful change in vesicle size, loss of drug, or alteration of their release profiles.^(32,33)^ These liposomal ciprofloxacin formulations were later modified with the addition of surfactant to generate formulations with faster release properties.^(20)^ These modified liposomes with faster release properties were also robust to the nebulization process with retention of vesicle size and encapsulated drug.^(21)^

More recently, we developed methods to transform the encapsulated drug into nanocrystalline form and attenuate its release rate [Cipolla, unpublished data]. The combination of these two innovations may provide a means to adjust the release behavior of a liposomal formulation to individualize treatment—to provide faster release and thus higher local drug concentrations in the lung (e.g., to exceed the pathogen's MIC) or slower/delayed release (e.g., to ensure drug is present when liposomes are taken up by infected macrophages).

In these studies the liposomal ciprofloxacin formulations containing 0.1% polysorbate 20 were frozen, and upon thawing the encapsulated drug was transformed into nanocrystalline form as verified by cryo-TEM imaging (Fig. 2B, C, and E). There was also a loss of ∼20% encapsulated drug as a consequence of the freeze-thaw procedure (Table 1). Formulations without polysorbate 20 did not lose any encapsulated drug after freeze-thaw but resulted in significantly larger vesicles to accommodate the longer drug crystals [Cipolla, unpublished data].

Preliminary studies on the formulations comprised of elongated liposomes containing nanocrystalline drug suggested they may be less robust to nebulization. In contrast, the mean vesicle size of the liposome formulations containing 0.1% polysorbate 20 increased by only 5–10 nm after freeze-thaw (Table 1), indicating that those formulations were not materially larger than the control CFI liposomes, and thus were more likely to be robust to the nebulization process. Formulations containing lower concentrations of polysorbate 20 (e.g., 0.05%) resulted in more elongated vesicles, while formulations containing higher concentrations of polysorbate 20 (e.g., 0.2%) had lower drug encapsulation following freeze-thaw [Cipolla, unpublished data]. The choice to use 0.1% polysorbate 20 was made to balance these two competing factors: to maximize the encapsulated drug and minimize the increase in vesicle size following freeze-thaw and thus retain vesicle stability to nebulization.

The presence of the surfactant led to inefficient jet nebulization due to foaming in the nebulizer. Thus, mesh nebulization was chosen for these studies. Mesh nebulization was successful at producing a respirable aerosol with a VMD of 3.6–3.9 μm and a GSD of ∼1.7 by laser diffraction for the CFI nanocrystalline formulations. Characterization of the pH 4.9 nanocrystalline CFI formulation by cascade impaction provided comparable aerosol droplet size values with an MMAD of 3.9 μm and a GSD of 1.45 (Fig. 4). Because the formulation density is near unity, it is not unexpected that there is close agreement between the values for VMD and MMAD. The MMAD for the nanocrystalline CFI formulation is similar to that obtained for the control (Fig. 4) and the VMD value is similar to that reported previously for the liposomal ciprofloxacin formulations containing surfactant which possessed faster release profiles (VMD of 3.7 μm and GSD of 1.7).^(21)^

The particle sizing experiment was followed by aerosol characterization studies to assess whether the liposome vesicles were compromised by nebulization. After nebulization, the mean vesicle size increased by 5–10 nm and the amount of encapsulated drug was reduced by ∼5% for the formulations between pH 4.9 to 5.9 (Table 2). This suggests that a small fraction of the liposomes may have been disrupted by the nebulization process releasing some of their encapsulated drug.

However, as evidenced by the cryo-TEM images (Fig. 1D, F), there was no apparent change in the appearance of the liposomes and the vast majority of the liposomes retained encapsulated drug in the nanocrystalline form. For the formulation at the lowest pH (4.6), there was a larger drop in the amount of encapsulated drug (Table 2). It is unclear why a lower external pH would lead to a greater loss in encapsulated drug. Cryo-TEM imaging was not performed on this formulation as it was removed from consideration going forward.

The ACI was found to be inappropriate for characterization of the liposome integrity for these liposome formulations due to liposome disruption on the ACI plates. For the control CFI formulation at pH 4.9 with 98% drug encapsulation, there was up to a 40% reduction in the percentage of encapsulated drug across the three stages of the ACI containing the majority of the deposited drug. Likewise, for the nanocrystalline liposomal formulation, the drug encapsulation was reduced to an even greater extent with reduction from ∼85% to between 9% and 64% encapsulated drug, suggesting that the ACI collection procedure is disrupting a large proportion of the liposome vesicles containing nanocrystalline ciprofloxacin. In contrast, using the simple collection system reported in this article, a reduction in percent encapsulation of only ∼5% was observed for the pH 4.9 formulation following nebulization, suggesting that only a small amount of liposome disruption is truly due to exposure to the mesh nebulization process.

The mechanism for vesicle disruption in the ACI may be due to impaction on the plates, exposure to the metal or air surfaces, or subsequent drying of the deposited liquid in the ACI. The latter mechanism may predominate as stage 4 was associated with the greatest loss of encapsulated drug and there was potential for more drying on stage 4 (than stage 2 or 3) due to the greater exposed liquid surface area relative to volume.

Use of cascade impactors for evaluation of the integrity of liposome formulations has been reported previously; for example, ∼30% loss of encapsulated drug was observed for liposomal amikacin when recovered in the ACI but this was attributed to disruption during nebulization and not an artifact of the collection process.^(29)^ In previous studies with liposomal ciprofloxacin, a biosampler containing recovery fluid was used to collect the nebulized liposomal ciprofloxacin formulations with no loss in liposome integrity.^(32,33)^ However, the biosampler method was also found to be inappropriate for the liposomal formulation containing nanocrystalline ciprofloxacin, presumably due to vesicle disruption due to exposure to shear and drying on the glass surfaces of the biosampler (unreported results).

Thus, for each new liposome formulation, which may be variably affected by dilution and drying on collection surfaces, an assessment must be made to determine the appropriate aerosol collection procedure. The compendial aerosol collection methods may be appropriate for routine QC analysis of the majority of formulations but may be inappropriate, as in this case, for characterization of more sophisticated formulations or unique products.

The presence of the drug nanocrystals within the liposomes led to slower release in the IVR assay immediately after freeze-thaw compared to that for the unfrozen control after frozen storage for 1 day (Fig. 3A) or 3 months (Fig. 3B). It is unclear why the samples at pH 4.9 and 5.3 had slightly slower release profiles after 3 months frozen storage compared to 1 day storage, presumably indicating more extensive nanocrystalline drug, but this may have been due to simple experimental variability in sample preparation or experimentation.

After mesh nebulization, the release profiles of the collected aerosol samples were substantially unchanged for the pH 4.9 and 5.3 formulations (Fig. 5) consistent with the continued presence of drug nanocrystals within the liposomes. A 90-minute delay in use of the thawed nanocrystalline formulation had no effect on its release profile (Fig. 6). However, 6 weeks storage at room temperature led to a change in the release profile, with a release profile intermediate between that of the unfrozen control and the slower releasing nanocrystalline formulation (Fig. 6). This suggests that a portion of the vesicles containing nanocrystalline drug may have reverted to a faster release profile more consistent with that for the unfrozen control. This may have been due to changes in the nanocrystalline state, or the permeability or integrity of the liposome vesicles. There may be other explanations as well.

Because ciprofloxacin is poorly soluble at neutral pH, the external pH of the standard liposome formulation at ∼6.0 was reduced to 5.3, 4.9, and 4.6 to improve the likelihood that the released ciprofloxacin would continue to remain in solution during extended room temperature storage. If the formulation were to be used immediately after thawing, then a change in pH might be unnecessary. The pH 4.6, 4.9, and 5.3 formulations retained good appearance after storage for 6 weeks at room temperature, in contrast to the pH 5.9 preparation that formed ciprofloxacin crystals external to the liposomes within 72 hours. However, in practice it would still be advisable for the formulation to be kept in a frozen state and to be thawed just prior to an expectation for use by the patient.

## Conclusions

A liposomal ciprofloxacin formulation containing 12.5 mg/mL ciprofloxacin, 90 mg/mL sucrose, and 0.1% polysorbate 20, over a pH range from 4.6 to 5.9 was stored frozen for up to 3 months. Upon thawing, the encapsulated drug was transformed to a nanocrystalline form, resulting in a slow-release profile *in vitro*. These modified formulations were nebulized using a vibrating mesh device to produce aerosols in the respirable size range, appropriate for delivery to the airways to treat lung disease.

While there were small changes in the encapsulation state and vesicle size following mesh nebulization, these formulations retained drug nanocrystals within the liposome vesicles as confirmed by cryo-TEM imaging. They also retained their attenuated IVR profile compared to the unmodified control liposomal ciprofloxacin formulation. These *in vitro* experiments, if confirmed in future *in vivo* studies, suggest that it may one day be possible to personalize inhaled therapy to individuals by transformation of the physical state of a liposomal drug following freeze-thaw, leading to a modified encapsulation state and IVR properties.
